# Combinations comprising dual β-lactams and a β-lactamase inhibitor achieve optimal synergistic inhibition of *Mycobacterium abscessus* growth

**DOI:** 10.1128/aac.00127-25

**Published:** 2025-08-14

**Authors:** Binayak Rimal, Yi Xie, Chandra M. Panthi, Kaylyn L. Devlin, Kimberly E. Beatty, Gyanu Lamichhane

**Affiliations:** 1 Division of Infectious Diseases, Department of Medicine, Johns Hopkins University School of Medicine 1500, Baltimore, Maryland, USA; 2 Department of Chemical Physiology and Biochemistry, Oregon Health Sciences University 6684, Portland, Oregon, USA; 3 Center for Nontuberculous Mycobacteria and Bronchiectasis, Johns Hopkins University School of Medicine 1500, Baltimore, Maryland, USA; City St George's, University of London, London, United Kingdom

**Keywords:** *Mycobacterium abscessus*, β-lactams, β-lactamase, β-lactamase inhibitor, dual β-lactam, dual β-lactamase inhibitor

## Abstract

The historical model, which posits that β-lactams inhibit bacterial growth while β-lactamase inhibitors (BLIs) merely protect β-lactams from enzymatic degradation, fails to fully explain their activity against *Mycobacterium abscessus* (*Mab*). This study demonstrates that synergistic effects extend beyond the traditional one β-lactam + one BLI paradigm, refuting the oversimplified mechanistic framework. First, β-lactam-based BLIs such as clavulanic acid, sulbactam, and tazobactam exhibit intrinsic antibacterial activity against *Mab*. These agents synergized not only with β-lactams but also with one another, undermining their historical classification as mere β-lactamase inhibitors. The data indicate that their activity is not limited to inhibiting β-lactamases but extends to directly targeting critical bacterial processes. Second, dual β-lactam combinations exhibit synergism against *Mab* even in the absence of BLIs. For example, despite being rapidly hydrolyzed by the native β-lactamase Bla_Mab_, amoxicillin demonstrates strong synergism with β-lactams such as imipenem or ceftaroline. This suggests that the second β-lactam either acts as a functional BLI surrogate or targets complementary pathways. Supporting this, experiments using penicillin- and carbapenem-based probes revealed that β-lactams bind to multiple *Mab* proteins simultaneously, reinforcing the idea that their synergy arises from targeting complementary essential proteins. Finally, triple combinations comprising dual β-lactam and one BLI, such as amoxicillin + ceftaroline + avibactam, achieved very high synergy, underscoring the complementary roles of dual β-lactams and BLIs. The evidence in this study necessitates a revised model that can more accurately explain the activities of β-lactams and BLIs and underscores the potential for optimizing β-lactam/BLI regimens against *Mab*.

## INTRODUCTION

The peptidoglycan, a key structural component of the bacterial cell wall, is the target for more than 50% of antibiotic prescriptions today, specifically those in the β-lactam class (1). The traditional model of β-lactam action was developed largely based on studies of a few model organisms, including *Escherichia coli, Staphylococcus aureus, and Bacillus subtilis*. According to this model, β-lactams exert their antibacterial effects by inhibiting enzymes essential for peptidoglycan synthesis, such as DD-transpeptidases (DDT, also known as penicillin-binding proteins or PBPs) (2). These enzymes are required for bacterial cell survival, growth, and division.

Bacteria commonly develop resistance to β-lactams through the production of β-lactamases, enzymes that hydrolyze and inactivate β-lactams (3). To counteract this, β-lactamase inhibitors (BLIs) are used in combination with β-lactams to treat infections caused by bacteria that produce β-lactamases. In this traditional model, BLIs are believed to only inhibit β-lactamases, thereby protecting the β-lactam, which retains its antibacterial activity in these combination treatments (4).

This model was challenged by evidence that demonstrated the presence of a new enzyme class that also catalyzes peptidoglycan synthesis but via a different mechanism than DDTs (5). While DDTs crosslink peptide side chains at 4→3 positions in the peptidoglycan, a distinct enzyme class forms crosslinks at 3→3 positions. This enzyme class, named LD-transpeptidase (LDT), was discovered by Mainardi et al. in a clinical isolate of *Enterococcus faecium* resistant to ampicillin, a penicillin-class β-lactam (6). In this isolate, resistance was due to LDT activity rather than β-lactamases or mutations in DDTs. Further studies showed that while penicillin-class β-lactams are less effective against LDTs, carbapenems—a different subclass of β-lactams—are potent inhibitors of these enzymes (7–9).

The organism used in this study, *Mycobacterium abscessus* (*Mab*), employs both LDTs and DDTs for peptidoglycan synthesis, with the majority of the linkages generated by the LDTs (10). In a related and more commonly known organism, *Mycobacterium tuberculosis* (*Mtb*), LDTs also generate the majority of peptidoglycan crosslinkages (11–13). LDTs are not unique to mycobacteria; they are found in several clinically important bacteria, including *Enterobacter cloacae, E. coli, Pseudomonas aeruginosa, Klebsiella pneumoniae,* and *Clostridioides difficile* (8, 14–17). Although 3→3 linkages in the peptidoglycan of *Mtb* and *E. coli* were first reported in 1974 and 1987, respectively (18, 19), the enzymatic basis for these linkages was overlooked because the traditional model emphasized DDTs as the sole source of peptidoglycan crosslinking.

The presence of both LDTs and DDTs in *Mab*, along with their differing susceptibilities to various β-lactam subclasses, led to the hypothesis that combining two β-lactam antibiotics (also known as dual β-lactams) targeting both enzyme classes may produce synergistic antibacterial effects (8). Indeed, independent research has confirmed this hypothesis, showing mechanistic evidence of synergy between β-lactams with distinct chemical structures (8, 9, 20). This synergy demonstrates the presence of multiple non-redundant proteins that are bound by β-lactams.

The *Mab* genome encodes five LDTs, multiple DDTs, and several putative DDTs based on sequence homology (17, 21). Since the catalytic sites of LDTs and DDTs are chemically and structurally distinct, no single β-lactam may effectively inhibit all these enzymes (22). Additionally, the *Mab* genome encodes at least one β-lactamase, Bla_Mab_ (gene locus MAB_2875) (23, 24). In *Mtb*, in addition to its native β-lactamase, BlaC (gene locus Rv2068c), several DDTs also inactivate β-lactams (25), highlighting the ability of several peptidoglycan synthesis enzymes to inactivate β-lactams in addition to those enzymes that have been traditionally believed to be the only source of β-lactamase activity.

Based on the evidence that multiple LDTs, DDTs, and β-lactamases exist in *Mab,* in this study, we hypothesized that a combination comprising multiple β-lactams and BLIs would exhibit synergistic effects against *Mab*. The rationale behind this hypothesis is that the presence of multiple LDTs, DDTs, and β-lactamases necessitates the use of multiple β-lactams and BLIs to simultaneously inhibit different targets in peptidoglycan synthesis, thereby effectively blocking the entire pathway. This hypothesis challenges the traditional paradigm, which relies on combinations of only one β-lactam and one BLI in clinical treatments of *Mab* infection (24, 26). If proven correct, this approach could demonstrate untapped potential in using β-lactams and BLIs in novel combinations to treat *Mab* and other bacterial infections where multiple LDTs, DDTs, and β-lactamases are critical to disease progression. Therefore, the overall aim of this study was not only to assess if combinations of multiple β-lactams and BLIs are more potent than dual β-lactams but also to generate proof-of-concept that can support future studies to develop more potent combinatorial treatments with β-lactams and BLIs.

To test this hypothesis, we evaluated and compared the potencies of combinations of clinically relevant β-lactams from all three subclasses and BLIs from known classes against *Mab* using the checkerboard assay (27). This assay generates the fractional inhibitory concentration index (FICI), which represents the summation of the inhibition of microbial growth contributed by each drug in the combination. A stringent interpretation of the FICI was used, with FICI of ≤0.5 indicating synergy, >0.5–<4 indicating indifference, and >4 indicating antagonism (28). An FICI of ≤0.5 results when two agents in the combination exist at only <0.25× their respective minimum inhibitory concentration (MIC) and yet inhibit the growth of the microbe.

## RESULTS

### Imipenem exhibits synergy with most β-lactams in inhibiting *Mab* growth *in vitro*

**Fig 1:**
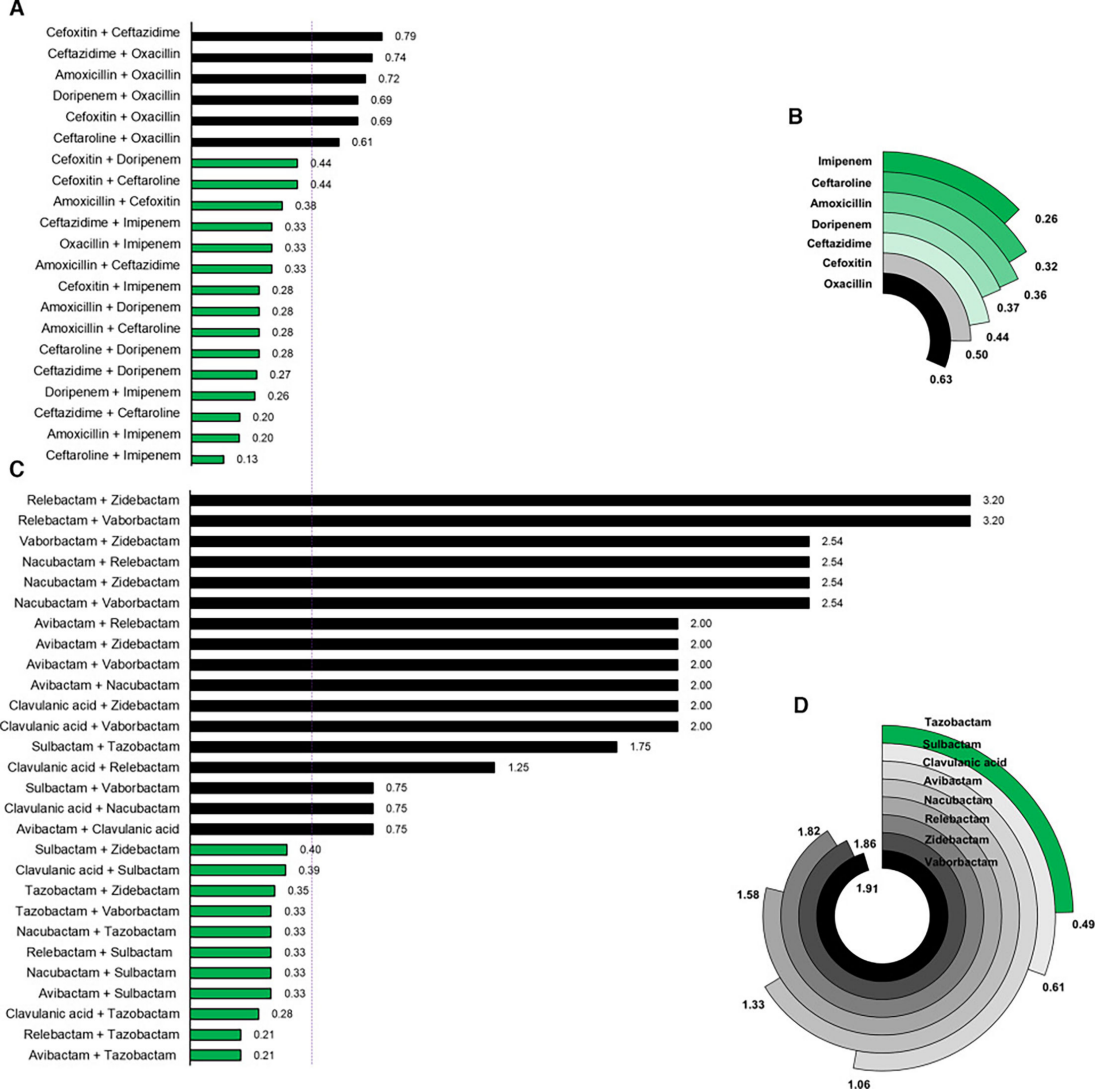
Activities of dual β-lactams or dual β-lactamase inhibitors against *M. abscessus*. (**A**) Fractional inhibitory concentration indexes of 21 dual β-lactams arising from seven β-lactams are shown. A dotted line at FICI of 0.5 demarks combinations that exhibited synergy (shown in green) and those that lacked synergy (shown in black). In total, 71% of dual β-lactams exhibited synergy. (**B**) The mean FICIs for dual β-lactams sharing one common β-lactam, listed on the left side of this figure, are shown. (**C**) FICIs of 28 dual BLIs arising from eight BLIs are shown. Synergy was observed in 39% of the dual BLIs. (**D**) The mean FICIs for dual BLIs sharing one common BLI are shown.

To assess the activity of different dual β-lactam combinations, seven β-lactams representing three widely used subclasses were evaluated. These included amoxicillin and oxacillin from the penicillin subclass; cefoxitin, ceftazidime, and ceftaroline from the cephalosporin subclass; and doripenem and imipenem from the carbapenem subclass. The MIC of each β-lactam against *Mab* was determined to establish activity arising from each individual agent (Table S1). Whether 21 dual β-lactams that comprise all possible combinatorial pairs from the seven β-lactams exhibit synergism, indifference, or antagonism in inhibiting *Mab* growth was assessed by determining their FICI. The FICIs for 15 dual β-lactams (71%) were ≤0.5, indicating synergistic effects in inhibiting *Mab* growth (Fig. 1A). The mean FICI for combinations sharing one common β-lactam was calculated to identify the β-lactams that contributed most to synergism (Fig. 1B). The dual β-lactams containing imipenem had the lowest mean FICI (0.26), demonstrating imipenem as the most common β-lactam in synergistic dual β-lactams. Therefore, the β-lactam that is most likely to exhibit synergy against *Mab* when combined with another β-lactam is imipenem. In contrast, dual β-lactams with oxacillin had the highest mean FICI (0.63), indicating that oxacillin exhibits synergy with the fewest β-lactams to inhibit *Mab* growth.

### Tazobactam synergizes with most β-lactamase inhibitors against *Mab* growth *in vitro*

Similarly, to determine the activities of dual BLI combinations (2BLI) against *Mab* growth, eight agents classified as BLIs were considered. Among them, clavulanic acid, sulbactam, and tazobactam belong to the β-lactam chemical class. Avibactam, nacubactam, relebactam, and zidebactam are non-β-lactam bicyclic agents. Vaborbactam, also a non-β-lactam, is a boronic acid agent (29). MICs of each BLI (Table S1) and FICIs of 28 dual BLIs arising from the BLI were determined against *Mab*. Eleven of these dual BLIs (39%) showed synergism, with FICI values ≤ 0.5 (Fig. 1C). Surprisingly, one of the agents in all synergistic dual BLIs is a β-lactam-based BLI such as clavulanic acid, sulbactam, or tazobactam. None of the dual BLIs composed entirely of non-β-lactam BLIs exhibited synergism. To identify the BLI that most frequently contributed to synergism, the mean FICI of pairs sharing one common BLI was calculated. Dual BLIs containing tazobactam had the lowest mean FICI (0.49), indicating its ability to exhibit synergy with the greatest number of BLIs in inhibiting *Mab* growth (Fig. 1D). Conversely, pairs with vaborbactam had the highest mean FICI, highlighting its ability to exhibit synergism with a limited number of BLIs.

### Several β-lactam + β-lactamase inhibitor combinations exhibit synergism against *Mab* growth

**Fig 2:**
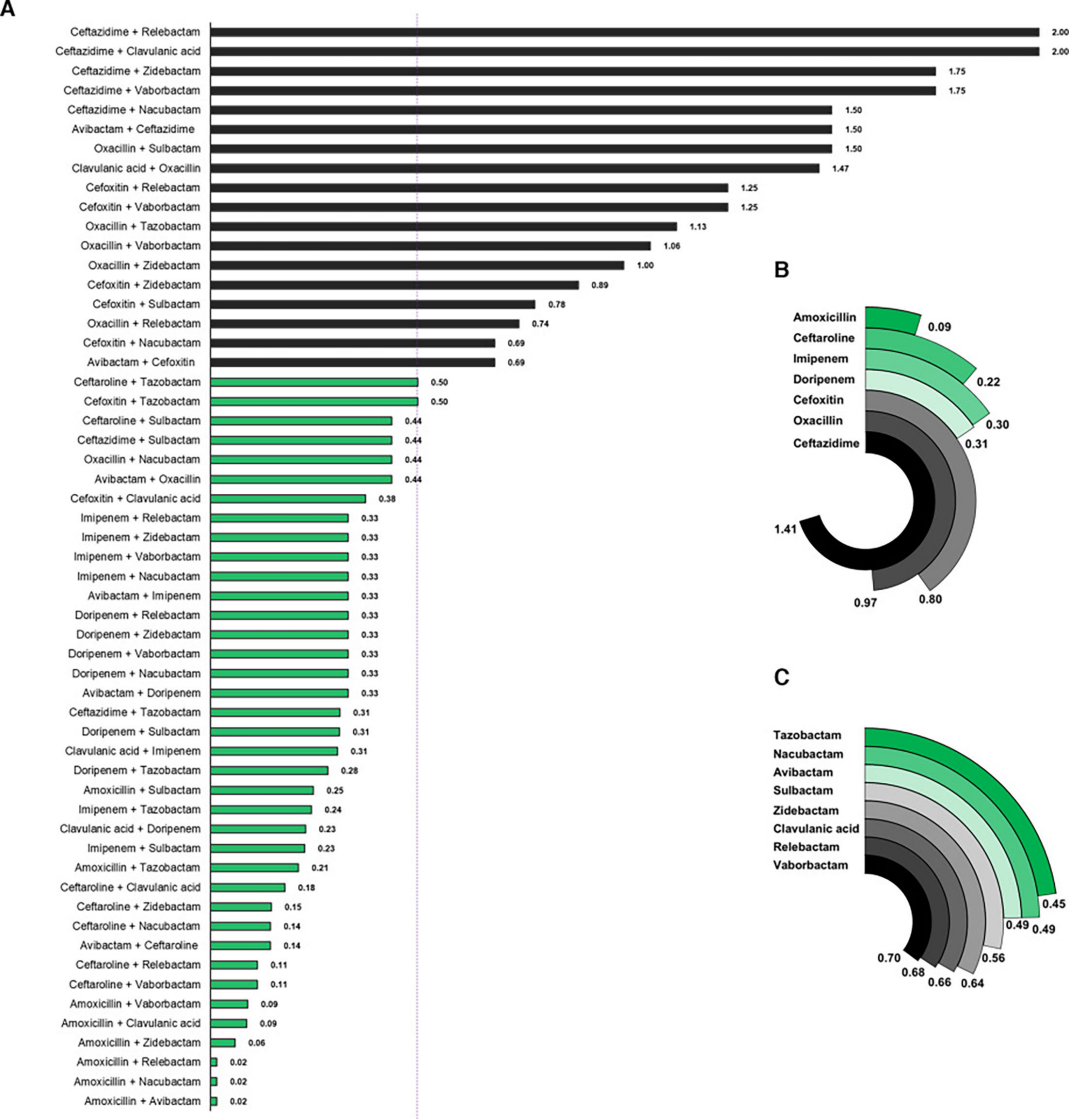
Activities of combinations comprising one β-lactam and one β-lactamase inhibitor (1B + 1BLI) against *M. abscessus*. (**A**) Fractional inhibitory concentration indexes of 56 1B + 1BLI arising from seven β-lactams and eight BLIs are shown. A dotted line at FICI of 0.5 demarks combinations that exhibited synergism (shown in green) and those that lacked synergism (shown in black). In total, 68% of 1B + 1BLI combinations exhibited synergy. (**B**) The mean FICIs for 1B + 1BLI combinations sharing one common β-lactam, listed on the left side of the figure, are shown. (**C**) The mean FICIs for 1B + 1BLI combinations sharing one common BLI are shown.

To identify β-lactam and BLI combinations that exhibit synergism in inhibiting *Mab* growth, the FICIs of 56 unique combinations comprising one β-lactam and one BLI (1B + 1BLI)—derived from the same seven β-lactams and eight BLIs considered above—were determined. Among these, 38 pairs (68%) had an FICI ≤ 0.5, indicating a synergistic effect in inhibiting *Mab* growth (Fig. 2A). To pinpoint which β-lactams were most synergistic across different BLIs, the mean FICI of each β-lactam paired with all BLIs was calculated. Amoxicillin had the lowest mean FICI (0.09) and exhibited synergism with all BLIs (Fig. 2B). Ceftaroline, imipenem, and doripenem also had mean FICIs below 0.5, indicating synergy with several BLIs. In contrast, ceftazidime, oxacillin, and cefoxitin showed synergy with the fewest BLIs, with ceftazidime being synergistic with only two. In summary, the β-lactams ranked by their synergy with BLIs in inhibiting *Mab* growth *in vitro* as follows: amoxicillin > ceftaroline > imipenem > doripenem > cefoxitin > oxacillin > ceftazidime.

To identify the BLIs that exhibit synergism with most β-lactams, the mean FICIs of each BLI paired with various β-lactams were calculated (Fig. 2C). Combinations containing tazobactam, nacubactam, or avibactam had mean FICIs < 0.5, indicating that these BLIs synergize with a broader range of β-lactams compared to the other BLIs. However, the differences in the number of β-lactams with which each BLI exhibited synergy were not sufficiently distinct to inform prioritization of one BLI over another.

### β-Lactamase inhibitors exhibit synergism with dual β-lactams against *Mab*

We hypothesized that the anti-*Mab* activity of synergistic dual β-lactams could be further enhanced by the addition of a BLI. This hypothesis is based on the presence of multiple proteins encoded by *Mab* that serve as targets for both β-lactams and BLIs (7, 9, 10). To test this hypothesis, we evaluated *Mab* growth in the presence of synergistic β-lactam pairs combined with a BLI and calculated the FICIs of the combinations.

Among the 15 synergistic dual β-lactams identified above and eight BLIs, all 120 unique combinations of dual β-lactams + one BLI (2B + 1BLI) were assessed. A checkerboard assay was conducted, with the dual β-lactams on one axis and a BLI on the other. Out of the 120 combinations tested, 110 (92%) showed FICI values ≤ 0.5, indicating that BLIs can indeed enhance the effectiveness of the majority of dual β-lactams in inhibiting *Mab* growth (Table 1).

**TABLE 1:** Activities of combinations comprising a dual β-lactam and a β-lactamase inhibitor against *M. abscessus* ATCC 19977^*a*^

Dual β-lactam	Sulbactam	Clavulanic acid	Tazobactam	Avibactam	Nacubactam	Vaborbactam	Zidebactam	Relebactam	Mean FICI
Amoxicillin + ceftaroline	**0.186**	**0.069**	**0.143**	**0.016**	**0.023**	**0.050**	**0.075**	**0.023**	0.073
Amoxicillin + imipenem	**0.171**	**0.084**	**0.126**	**0.016**	**0.098**	**0.035**	**0.098**	**0.098**	0.091
Amoxicillin + ceftazidime	**0.186**	**0.148**	**0.186**	**0.098**	**0.098**	**0.100**	**0.140**	**0.098**	0.132
Ceftaroline + doripenem	**0.133**	**0.122**	**0.143**	**0.145**	**0.145**	**0.145**	**0.145**	**0.145**	0.140
Amoxicillin + doripenem	**0.290**	**0.102**	**0.264**	**0.098**	**0.098**	**0.094**	**0.132**	**0.091**	0.146
Cefoxitin + ceftaroline	**0.232**	**0.143**	**0.132**	**0.145**	**0.145**	**0.145**	**0.155**	**0.145**	0.155
Ceftazidime + ceftaroline	**0.184**	**0.167**	**0.176**	**0.145**	**0.145**	**0.145**	**0.165**	**0.145**	0.159
Amoxicillin + cefoxitin	**0.347**	**0.176**	**0.259**	**0.098**	**0.098**	**0.100**	**0.129**	**0.098**	0.163
Imipenem + oxacillin	**0.284**	**0.217**	**0.171**	**0.207**	**0.207**	**0.207**	**0.332**	**0.207**	0.229
Ceftaroline + imipenem	1.037	**0.223**	**0.297**	**0.145**	**0.145**	**0.207**	**0.207**	**0.155**	0.302
Cefoxitin + doripenem	**0.367**	**0.280**	**0.311**	**0.312**	**0.332**	**0.332**	**0.332**	**0.332**	0.325
Ceftazidime + doripenem	**0.417**	**0.273**	**0.327**	**0.338**	**0.332**	**0.332**	**0.332**	**0.332**	0.335
Doripenem + imipenem	**0.311**	**0.262**	**0.311**	**0.332**	**0.332**	**0.332**	**0.500**	**0.332**	0.339
Cefoxitin + imipenem	**0.332**	**0.218**	**0.332**	**0.332**	0.749	0.749	0.749	0.749	0.526
Ceftazidime + imipenem	**0.354**	**0.313**	**0.438**	1.325	1.500	1.583	1.187	1.469	1.021

^*a*^ The fractional inhibitory concentration indexes of each dual β-lactam + β-lactamase inhibitor combination are shown. FICIs ≤ 0.5, indicating synergism, are underlined and bolded. The last column lists the mean FICI for each dual β-lactam when combined with a β-lactamase inhibitor.

Among these, the dual β-lactams (amoxicillin + ceftaroline) and (amoxicillin + imipenem) showed the highest synergy when combined with avibactam, achieving FICI values of 0.016 for each combination. We calculated the mean FICIs for each dual β-lactam to identify those that synergized most frequently with different BLIs (Table 1). Based on the mean FICI values, the dual β-lactams ranked by their synergistic potential when combined with a BLI as follows: (amoxicillin + ceftaroline) > (amoxicillin + imipenem) > (amoxicillin + ceftazidime) > (ceftaroline + doripenem) > (amoxicillin + doripenem) > (cefoxitin + ceftaroline) > (ceftazidime + ceftaroline) > (amoxicillin + cefoxitin) > (imipenem + oxacillin) > (ceftaroline + imipenem) > (cefoxitin + doripenem) > (ceftazidime + doripenem) > (doripenem + imipenem) > (cefoxitin + imipenem) > (ceftazidime + imipenem). Notably, dual β-lactams containing amoxicillin were most frequently synergistic when paired with a BLI.

Among the 10 combinations that did not exhibit synergy, 4 (40%) were combinations of BLIs with (imipenem + cefoxitin), and 5 (50%) were combinations with (imipenem + ceftazidime). The addition of several BLIs to the dual β-lactams (imipenem + cefoxitin) and (imipenem + ceftazidime) failed to lower their FICI, perhaps due to the high level of synergy between imipenem and cefoxitin (FICI = 0.28) and imipenem and ceftazidime (FICI = 0.33) (Fig. 1A). This suggests that adding a BLI provides only marginal enhancement to the activities of these particular dual β-lactams.

### Adding a second β-lactamase inhibitor improves potencies of select β-lactam + β-lactamase inhibitor pairs

We evaluated whether the anti-*Mab* activity of synergistic dual BLIs could be enhanced by adding a β-lactam. Specifically, we tested *Mab* growth *in vitro* in the presence of combinations comprising one β-lactam and dual BLIs (1B + 2BLI). A total of 77 unique 1B + 2BLI combinations, derived from 7 β-lactams and 11 synergistic dual BLI identified above, were assessed. The FICI indicated synergy (FICI ≤ 0.5) in 60 (78%) of these combinations (Table 2).

**TABLE 2:** Activities of combinations comprising dual β-lactamase inhibitors and a β-lactam against *M. abscessus* ATCC 19977^*a*^

Dual β-lactamase inhibitor	Amoxicillin	Cefoxitin	Ceftazidime	Ceftaroline	Doripenem	Imipenem	Oxacillin	Mean FICI
Tazobactam + avibactam	**0.016**	**0.217**	**0.332**	**0.107**	**0.163**	0.749	**0.162**	0.249
Clavulanic acid + tazobactam	**0.143**	**0.311**	**0.290**	**0.176**	**0.176**	**0.332**	**0.332**	0.252
Tazobactam + nacubactam	**0.031**	**0.280**	**0.332**	**0.072**	**0.186**	0.749	**0.228**	0.268
Tazobactam + vaborbactam	**0.037**	**0.311**	**0.332**	**0.112**	**0.150**	0.749	**0.249**	0.277
Sulbactam + avibactam	**0.016**	**0.263**	0.749	**0.104**	**0.193**	0.749	**0.228**	0.329
Sulbactam + relebactam	**0.026**	**0.258**	0.749	**0.107**	**0.167**	0.749	**0.270**	0.332
Sulbactam + nacubactam	**0.031**	**0.258**	**0.367**	**0.104**	**0.227**	1.249	**0.367**	0.372
Tazobactam + zidebactam	**0.086**	**0.354**	0.749	**0.143**	**0.186**	0.749	**0.347**	0.373
Clavulanic acid + sulbactam	**0.159**	**0.354**	**0.290**	**0.176**	**0.232**	0.687	0.749	0.378
Sulbactam + zidebactam	**0.125**	**0.438**	0.749	**0.143**	**0.181**	0.749	**0.396**	0.397
Tazobactam + relebactam	1.070	**0.280**	**0.332**	**0.072**	**0.186**	0.749	1.070	0.537

^*a*^ The fractional inhibitory concentration indexes of each dual β-lactamase inhibitor + β-lactam combination are shown. FICIs ≤ 0.5, indicating synergism, are underlined and bolded. The last column lists the mean FICI for each dual β-lactamase inhibitor when combined with a β-lactam.

This synergy rate marks a 10% increase over the 68% observed with 1B + 1BLI combinations, suggesting that many 1B + 1BLI pairs gain additional activity when a second BLI is added. For all β-lactams except imipenem, the addition of a second BLI almost always led to a synergistic 1B + 2BLI combination. Amoxicillin demonstrated strong synergy with dual BLIs (lowest average FICI), with the exception of the relebactam + tazobactam pair. Cefoxitin, ceftaroline, and doripenem consistently exhibited synergy with all tested dual BLI combinations. Conversely, imipenem generally showed no additional activity (FICI > 0.5) when a second BLI was added, except in the combination of clavulanic acid + tazobactam.

### Triple combinations comprising dual β-lactams + one BLI exhibit added synergy against *Mab*

As described above, the addition of a BLI to dual β-lactams or combinations of 1β-lactam + 1BLI leads to synergy in inhibiting *Mab* growth *in vitro*. This suggests that these triple combinations inhibit complementary targets in *Mab*. However, experiments described so far have not tested if these triple combinations saturate the available β-lactam and BLI targets in *Mab*.

To explore this idea, we hypothesized that combining four agents—two β-lactams and two BLIs (2B + 2BLI)—may saturate available targets and manifest as enhanced synergism. To test this hypothesis, we evaluated the activities of 165 unique 2B + 2BLI combinations derived from 15 synergistic dual β-lactams and 11 synergistic dual BLIs using the checkerboard assay. For 2B + 2BLI to achieve synergy, each component would have to be present at ≤1/16-fold their respective MICs for the net FICI to be ≤0.5. Of the 165 2B + 2BLI combinations, 130 (79%) were not synergistic. The FICI was ≤0.5 for 35, all of which included amoxicillin as the common β-lactam (Table S2). Therefore, except for 2B + 2BLI combinations that included amoxicillin, combinations comprising three agents, whether dual β-lactam + β-lactamase inhibitor or a β-lactam + dual β-lactamase inhibitor, may be sufficient to saturate available targets in *Mab*.

### β-lactam probes bind to multiple proteins in *Mab*

**Fig 3:**
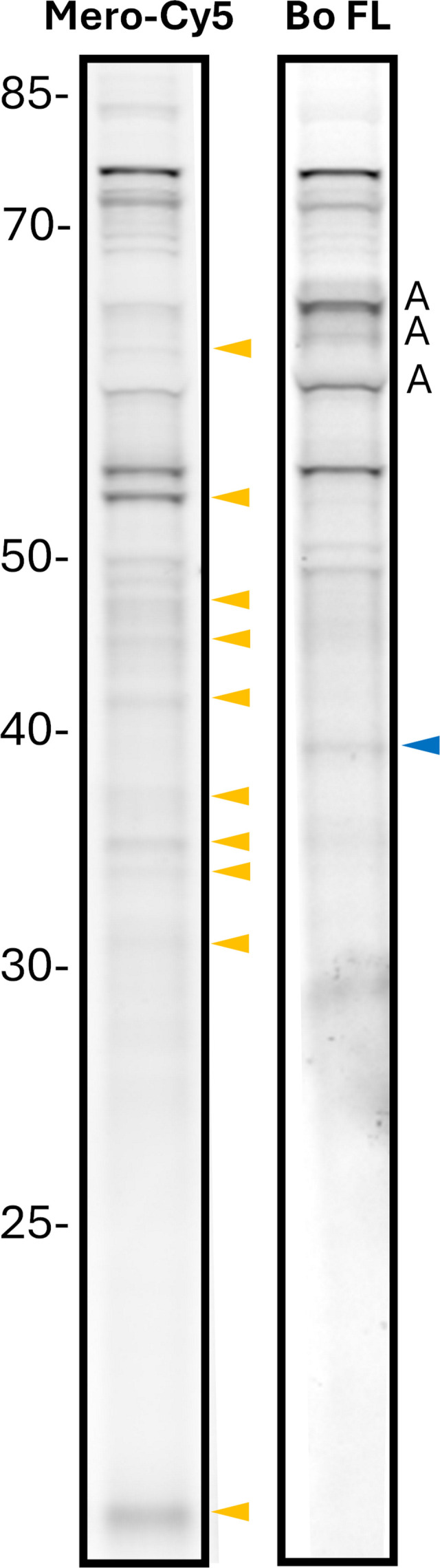
β-lactam probes bind multiple proteins in *Mab* lysates. *Mab* whole-cell lysate was treated with meropenem-Cy5 (Mero-Cy5) or Bocillin-FL (BoFL), resolved using SDS-PAGE, and imaged. Three autofluorescent bands (A) were observed in the green channel (BoFL), but none were observed in the Cy5 channel. Targets uniquely identified by Mero-Cy5 are marked with a yellow arrow, while those identified by BoFL are marked with a blue arrow. The markers on the left denote the molecular weight of the proteins in kilodaltons.

The findings described above suggest that β-lactams and BLIs inhibit complementary targets in *Mab* to achieve synergy. However, the assays based on assessing *Mab* growth inhibition in the presence of β-lactams and BLIs do not directly confirm the binding of these agents to multiple targets in *Mab*. To generate more direct evidence of proteins in *Mab* that are bound by β-lactams, as a proof-of-concept, we labeled *Mab* lysates with a recently described red fluorescent meropenem-Cy5, a β-lactam of the carbapenem subclass (30). To represent the penicillin subclass, we used the commercially available green fluorescent Bocillin-FL probe. These probes were incubated with *Mab* whole-cell lysate, and the resulting mixtures were analyzed using SDS-PAGE (Fig. 3). Fluorescence scans of the gels reveal multiple bands, each corresponding to a unique protein bound by the probes. The meropenem probe revealed many enzymes not labeled by Bocillin-FL. While several proteins were bound by probes, variable band intensities suggest differential affinity of the β-lactam probes to the target proteins. These results provide direct evidence that there are many targets of β-lactams in *Mab* and that different β-lactam subclasses bind to multiple proteins in *Mab* but with distinct affinities and selectivities.

### Potential translational relevance

The use of a β-lactam in combination with a β-lactamase inhibitor is a well-established strategy in clinical practice for treating bacterial infections. However, the evidence presented here suggests that this approach may be underexploiting the full potential of these drugs in the context of *Mab*. Although the data highlight promising synergy between certain drug combinations, it remains uncertain whether the concentrations required for these effects are achievable in patients—an essential criterion for clinical applicability.

To evaluate the therapeutic potential of these synergistic combinations, we determined the MICs for each drug from the checkerboard assay data (Table 3). Our results show several combinations of dual β-lactams and β-lactam + BLI, where the MICs of individual drugs were reduced from clinically unviable levels to potentially therapeutic levels.

**TABLE 3:** Synergistic combinations of β-lactams, β-lactam + β-lactamase inhibitor, and β-lactamase inhibitor + β-lactamase inhibitor, along with their MICs when used alone or in combinations, are shown^*a*^

Synergistic combination drug A + drug B	MIC of a drug when used alone → MIC of the drug in combination (µg/mL)	
	Drug A	Drug B
Cefoxitin + doripenem	16 → 2	16 → 2
Cefoxitin + ceftaroline	16 → 4	512 → 64
Amoxicillin + cefoxitin	1,024 → 128	16 → 4
Ceftazidime + imipenem	512 → 4	8 → 2
Oxacillin + imipenem	1,024 → 8	8 → 2
Amoxicillin + ceftazidime	1,024 → 256	512 → 4
Cefoxitin + imipenem	16 → 2	8 → 1
Amoxicillin + doripenem	1,024 → 128	16 → 2
Amoxicillin + ceftaroline	1,024 → 128	512 → 64
Ceftaroline + doripenem	512 → 64	16 → 2
Ceftazidime + doripenem	512 → 32	16 → 1
Doripenem + imipenem	16 → 2	8 → 1
Ceftazidime + ceftaroline	512 → 32	512 → 64
Amoxicillin + imipenem	1,024 → 8	8 → 1
Ceftaroline + imipenem	512 → 8	8 → 1
Ceftaroline + tazobactam	512 → 128	1,024 → 128
Cefoxitin + tazobactam	16 → 4	1,024 → 256
Ceftaroline + sulbactam	512 → 128	1,024 → 128
Ceftazidime + sulbactam	512 → 64	1,024 → 256
Oxacillin + nacubactam	1,024 → 256	512 → 64
Oxacillin + avibactam	1,024 → 256	512 → 64
Cefoxitin + clavulanic acid	16 → 4	512 → 64
Imipenem + relebactam	8 → 2	1,024 → 8
Imipenem + zidebactam	8 → 2	512 → 4
Imipenem + vaborbactam	8 → 2	512 → 4
Imipenem + nacubactam	8 → 2	512 → 4
Imipenem + avibactam	8 → 2	512 → 4
Doripenem + relebactam	16 → 4	1,024 → 8
Doripenem + zidebactam	16 → 4	512 → 4
Doripenem + vaborbactam	16 → 4	512 → 4
Doripenem + nacubactam	16 → 4	512 → 4
Doripenem + avibactam	16 → 4	512 → 4
Ceftazidime + tazobactam	512 → 64	1,024 → 128
Doripenem + sulbactam	16 → 4	1,024 → 8
Imipenem + clavulanic acid	8 → 2	512 → 4
Doripenem + tazobactam	16 → 2	1,024 → 128
Amoxicillin + sulbactam	1,024 → 128	1,024 → 64
Imipenem + tazobactam	8 → 1	1,024 → 64
Doripenem + clavulanic acid	16 → 2	512 → 16
Imipenem + sulbactam	8 → 1	1,024 → 64
Amoxicillin + tazobactam	1,024 → 64	1,024 → 64
Ceftaroline + clavulanic acid	512 → 16	512 → 32
Ceftaroline + zidebactam	512 → 32	512 → 8
Ceftaroline + nacubactam	512 → 32	512 → 4
Ceftaroline + avibactam	512 → 32	512 → 4
Ceftaroline + relebactam	512 → 16	1,024 → 8
Ceftaroline + vaborbactam	512 → 16	512 → 4
Amoxicillin + vaborbactam	1,024 → 8	512 → 4
Amoxicillin + clavulanic acid	1,024 → 32	512 → 16
Amoxicillin + zidebactam	1,024 → 32	512 → 8
Amoxicillin + relebactam	1,024 → 8	1,024 → 8
Amoxicillin + nacubactam	1,024 → 8	512 → 4
Amoxicillin + avibactam	1,024 → 8	512 → 4
Sulbactam + zidebactam	1,024 → 256	512 → 32
Clavulanic acid + sulbactam	512 → 128	1,024 → 64
Tazobactam + zidebactam	1,024 → 256	512 → 8
Tazobactam + vaborbactam	1,024 → 256	512 → 4
Nacubactam + tazobactam	512 → 4	1,024 → 256
Relebactam + sulbactam	1,024 → 8	1,024 → 256
Nacubactam + sulbactam	512 → 4	1,024 → 256
Avibactam + sulbactam	512 → 4	1,024 → 256
Clavulanic acid + tazobactam	512 → 64	1,024 → 64
Relebactam + tazobactam	1,024 → 8	1,024 → 256
Avibactam + Tazobactam	512 → 4	1,024 → 128

^*a*^ The second and third columns display each drug’s MIC against *M. abscessus* ATCC 19977 when used alone, followed by an arrow (→) indicating the MIC change when used in the corresponding combination listed in column one. Clinical and Laboratory Standards Institute (CLSI) breakpoints (≤16 µg/mL, susceptible; 32–64 µg/mL, intermediate; and ≥128 µg/mL, resistant) for cefoxitin were used for all cephalosporins. Similarly, CLSI breakpoints (≤4 µg/mL, susceptible; 8–16 µg/mL, intermediate; and ≥32 µg/mL, resistant) for imipenem and meropenem were used for all carbapenems. Due to the lack of breakpoints for penicillins and β-lactamase inhibitors against *Mab*, their MICs are not interpreted.

For example, in the dual β-lactam combination of ceftazidime and doripenem, the MIC of ceftazidime decreased from 512 to 32 μg/mL, and doripenem’s MIC fell from 16 to 1 μg/mL. While a ceftazidime MIC of 512 µg/mL is considered clinically ineffective, 32 µg/mL is categorized as intermediate susceptibility according to Clinical and Laboratory Standards Institute (CLSI) guidelines (inferred from the cefoxitin breakpoint due to the absence of one for ceftazidime) (31), and thus may be therapeutically meaningful. Similarly, doripenem’s MIC decreased to a level considered susceptible, supporting its potential utility in combination therapy.

In β-lactam + BLI pairings, combinations such as ceftaroline with relebactam demonstrate clinically relevant synergy. MICs dropped from 512 and 1,024 µg/mL for ceftaroline and relebactam, respectively, to 16 and 8 µg/mL—levels that are likely to be achievable in clinical settings. Another effective combination, amoxicillin with avibactam, reduced MICs from 1,024 and 512 μg/mL to 8 and 4 µg/mL, respectively, again reaching clinically useful concentrations.

In contrast, BLI + BLI combinations do not appear clinically promising. Although these combinations also resulted in reduced MICs, the final concentrations remained relatively high. Moreover, the lack of CLSI breakpoints for BLIs limits our ability to interpret their clinical relevance. These findings suggest that while the BLI + BLI strategy shows a consistent trend of MIC reduction, it is unlikely to translate into a viable clinical option.

## DISCUSSION

The historical model describing the mechanistic basis of β-lactams and BLIs posits that β-lactams inhibit microbial growth, while BLIs serve solely to protect β-lactams from degradation by β-lactamases (4). This framework has guided the development of combinations of one β-lactam and one BLI for treating bacterial infections. However, evidence from this study demonstrates that this model does not fully apply to *Mab*. Notably, numerous instances of synergism were observed against *Mab* in combinations that deviate from the traditional one β-lactam–one BLI paradigm.

The first line of evidence is the synergistic activity of select dual β-lactams against *Mab*, even in the absence of a BLI (Fig. 1A). The relevance of β-lactamase activity to this synergy remains unclear. For example, amoxicillin is inactivated in the presence of the β-lactamase Bla_Mab_ (32), which explains its high MIC against *Mab* (Table S1). Despite this, amoxicillin exhibits strong synergism when paired with β-lactams such as imipenem, doripenem, cefoxitin, ceftazidime, or ceftaroline (Fig. 1A). This raises the question of whether the second β-lactam in these pairs acts as a functional BLI for amoxicillin. Notably, the multiple protein targets bound by meropenem-Cy5 and Bocillin-FL probes (Fig. 3) may hold the answer to this conundrum. It suggests that synergism of dual β-lactams is achieved by simultaneous inhibition of several complementary targets.

The second line of evidence challenges the traditional role of BLIs as β-lactamase inhibitors without intrinsic antibacterial activity. Synergistic dual BLIs were identified, particularly those involving β-lactam-based agents such as clavulanic acid, sulbactam, and tazobactam (Fig. 1C). These findings suggest that these agents may also inhibit essential targets in *Mab* beyond β-lactamase activity. For instance, in a prior study to assess β-lactam targets in *M. tuberculosis*, pre-treatment with clavulanic acid blocked labeling of its β-lactamase, BlaC, by meropenem-Cy5. However, labeling of several other proteins was also lost, suggesting that clavulanic acid inhibits multiple targets (30). Historically regarded as distinct from β-lactams, these BLIs may share antibacterial properties due to their chemical composition as β-lactams. For example, the sulbactam + durlobactam combination is an example of a dual BLI, recently approved by the FDA for bacterial pneumoniae, which likely inhibits proteins essential for cell survival in addition to inhibiting β-lactamases (33).

Tazobactam, frequently found in synergistic combinations (mean FICI = 0.49), appears to have a broader spectrum of activity against *Mab* targets compared to other BLIs. Previous studies indicate that tazobactam, sulbactam, and clavulanic acid do not inhibit Bla_Mab_ (23), implying the presence of additional proteins targeted by tazobactam. As tazobactam exhibited synergy with several β-lactams in our study, this evidence indicates the presence of β-lactamase activity in addition to Bla_Mab_ that is relevant to *Mab* growth and is inhibited by tazobactam. Conversely, vaborbactam demonstrated limited synergy, likely reflecting its limited specificity for *Mab*’s β-lactamase repertoire.

Adding a BLI to synergistic dual β-lactams provided variable enhancement, with the greatest synergy observed in amoxicillin-based combinations. For instance, imipenem-cefoxitin showed strong baseline synergy (FICI = 0.28), leaving limited room for improvement. A prior study that assessed the synergism of several dual β-lactams against 21 *Mab* clinical isolates found that imipenem + cefoxitin exhibited synergy against 100% of the isolates (34). In contrast, amoxicillin combinations exhibited greater enhancement, likely due to the restoring activity of BLIs by neutralizing enzymatic degradation. Notably, triple combinations such as amoxicillin + ceftaroline + avibactam achieved exceptional synergy (FICI = 0.016), underscoring the complementary roles of β-lactams and BLIs.

Synergism was also observed in combinations involving 1B + 2BLI, with 60 of 77 (78%) combinations achieving FICI ≤ 0.5—a 10% increase over 1B + 1BLI combinations. For example, amoxicillin + clavulanic acid + nacubactam exhibited synergy, likely due to complementary inhibition of multiple β-lactamase classes. In contrast, among 165 2B + 2BLI combinations, only those that contained amoxicillin produced synergy. This finding suggests redundancy of the fourth agent except for combinations containing amoxicillin.

**Fig 4:**
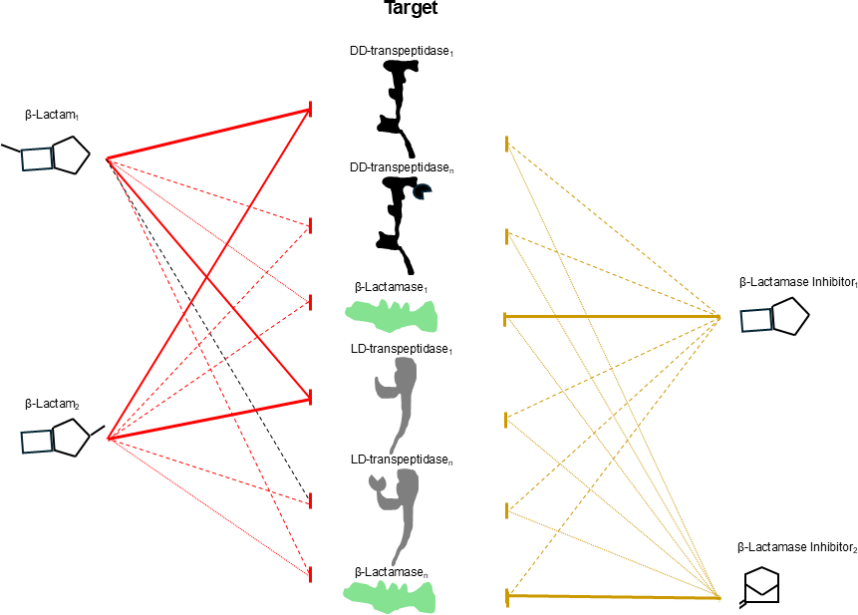
This model outlines the roles of β-lactams and β-lactamase inhibitors in targeting enzymes in *M. abscessus*. β-lactam_1_ and β-lactam_2_ represent two distinct β-lactams. They bind to an overlapping set of targets but exhibit varying efficiencies in inhibiting specific targets. Similarly, β-lactamase inhibitor_1_ and β-lactamase inhibitor_2_ represent two distinct β-lactamase inhibitors with each inhibiting overlapping targets but with varying efficiency. β-lactamase inhibitor_1_ is based on a β-lactam chemical structure, whereas β-lactamase inhibitor_2_ represents non-β-lactam-based structure. Target enzymes: DD-transpeptidase_1_ and DD-transpeptidase_*n*_ depict the first and the *n*th DD-transpeptidase. Similarly, LD-transpeptidase_1_ and LD-transpeptidase_*n*_ depict the first and the *n*th LD-transpeptidase, and β-lactamase_1_ and β-lactamase_*n*_ depict the first and the *n*th β-lactamase in *M. abscessus*. The thickness of the lines connecting β-lactams and β-lactamase inhibitors to their targets indicates the strength of inhibition.

Based on the emerging evidence from this study and prior research (8, 9, 26, 35–41), we propose a revised model to more accurately describe the activities of β-lactams and BLIs in *Mab* (Fig. 4). In this model, each β-lactam targets multiple proteins critical for peptidoglycan synthesis, with each β-lactam in a synergistic pair generating complementary inhibition required to achieve optimal inhibition of essential targets. Furthermore, agents historically classified as BLIs—particularly those with β-lactam-based structures such as clavulanic acid, sulbactam, and tazobactam—are likely not restricted to β-lactamase inhibition alone. Instead, our findings suggest that synergistic dual BLIs also target essential components of peptidoglycan synthesis, contributing directly to bacterial growth inhibition. This model describes how a β-lactam or a BLI can inhibit multiple targets, without suggesting that using more than three drugs—or combining a dual β-lactam with a BLI—would lead to enhanced inhibition of *Mab* growth.

Molecular mechanisms such as drug transport, efflux pump activity, and the metabolic state of the bacterial cell significantly influence the overall response of *Mab* to drugs including β-lactams and BLIs. These factors can affect intracellular drug concentrations, alter the expression of resistance determinants, and modulate the efficacy of antibacterial agents. Nonetheless, the current model is intentionally focused on a more defined aspect: the inhibition of β-lactamases and key enzymes involved in peptidoglycan synthesis directly targeted by β-lactams. By narrowing the scope, the model aims to provide mechanistic clarity regarding how β-lactams and BLIs interact with their primary enzymatic targets in *Mab*, recognizing that broader cellular processes—while critically important—introduce additional layers of complexity beyond the model’s immediate framework.

In conclusion, the evidence from this study challenges the simplistic view that β-lactams target PBPs and LDTs essential for peptidoglycan synthesis, while BLIs merely inhibit β-lactamases. Instead, both β-lactams and BLIs appear to inhibit complementary targets in *Mab*. These findings highlight the potential to optimize β-lactam/BLI regimens, including novel triple combinations, to treat *Mab* infections. Future research should explore these mechanisms further, paving the way for more effective therapeutic strategies. Several synergistic dual β-lactams and β-lactam + BLI combinations significantly reduce MICs of each component to clinically achievable levels, suggesting that multiple pairings hold strong potential for therapeutic application to treat *Mab* infection. *In vitro* models like the checkerboard assay offer valuable insights into the potential of drug combinations, but they do not always reliably predict dose or efficacy in a host. Therefore, it is essential to validate these findings using a relevant *in vivo* model.

## MATERIALS AND METHODS

### Bacterial strains, drugs, culture media, and *in vitro* growth conditions

*Mycobacterium abscessus* strain ATCC 19977, isolated in 1950, commonly included as a reference for *Mab* in laboratory settings, was used in this study (42). The strain was procured from the American Type Culture Collection (Manassas, VA, USA) and authenticated by genome sequencing (34). It was cultured in Middlebrook 7H9 broth (Difco, catalog no. 271310) supplemented with 0.5% glycerol, 10% albumin-dextrose-salt enrichment, and 0.05% Tween-80 at 37°C in an orbital shaker at 220 RPM as described (43). However, for all assays involving determination of drug activities, Middlebrook 7H9 broth supplemented with 0.5% glycerol and 10% albumin-dextrose-salt enrichment without Tween-80 was used in U-bottom 96-well plates with 350 µL capacity per well. Powdered form of drugs was purchased from commercial vendors as follows: amoxicillin (Sigma-Aldrich, catalog no. A8523), oxacillin (Sigma-Aldrich, catalog no. 28221), cefoxitin (Sigma-Aldrich, catalog no. C4786), ceftazidime (Sigma-Aldrich, catalog no. C3809), ceftaroline fosamil hydrate (Sigma-Aldrich, catalog no. SML3102), imipenem (Sigma-Aldrich, catalog no. I0160), doripenem (Sigma-Aldrich, catalog no. SML1220), sulbactam (Sigma-Aldrich, catalog no. S9701), tazobactam (Sigma-Aldrich, catalog no. T2820), clavulanic acid (BOC Sciences, catalog no. B0084-073058), avibactam (Sigma-Aldrich, catalog no. SBR00075), relebactam (MedChemExpress, catalog no. HY-16752), vaborbactam (MedChemExpress, catalog no. HY-19930), zidebactam (MedChemExpress, catalog no. 120859), and nacubactam (MedChemExpress, catalog no. 109008).

### Determination of minimum inhibitory concentration of a drug

The MICs of β-lactams, amoxicillin, oxacillin, cefoxitin, ceftazidime, ceftaroline fosamil hydrate, imipenem, and doripenem, and β-lactamase inhibitors, sulbactam, tazobactam, clavulanic acid, avibactam, relebactam, vaborbactam, zidebactam, and nacubactam, against *Mab* were determined using a broth microdilution assay in accordance with the Clinical and Laboratory Standards Institute guidelines (31). A 10 mg/mL stock solution of each drug was made by dissolving in dimethyl sulfoxide, which was used to make working solutions at lower dilutions in Middlebrook 7H9 broth supplemented with 0.5% glycerol and 10% albumin-dextrose-salt enrichment without Tween-80. Working drug solutions were diluted twofold serially in Middlebrook 7H9 broth to generate concentrations ranging from 512 to 0.06 μg/mL. Using *Mab* grown to the exponential phase, 10^5^ CFU of *Mab* were inoculated into each well of a 96-well culture plate, in a final volume of 200 µL per well. Two wells containing Middlebrook 7H9 broth only and two wells containing broth inoculated with 10^5^ CFU of *Mab* were included as negative and positive controls, respectively, and incubated at 30°C for 72 h without shaking in accordance with the CLSI guidelines (31). Growth or lack thereof of *Mab* was assessed using the Sensititre Manual Viewbox. The lowest concentration of the drug at which well suspension appeared identical to the well with broth only was recorded as the MIC of the drug. Two biological replicates with freshly grown *Mab* cultures and two technical replicates within each assay were performed. The final MIC was calculated as the mean of the MICs in biological and technical replicates.

### Determination of fractional inhibitory concentration

The checkerboard assay was used to determine the activities of combinations of drugs. This assay is a modification of the standard broth microdilution assay used for MIC determination and was performed as described (27). The most stringent guideline for interpreting FICI was used: an FICI of ≤0.5 was interpreted as synergy, >0.5–4 was interpreted as indifference, and >4 as antagonism (28).

### Determination of FICI for two drugs (B + B or BLI + BLI or B + BLI)

To determine the FICIs of two drugs, working solutions of the two drugs were added to Middlebrook 7H9 broth, each starting at fourfold MIC and serially diluted up to 1/64-fold MIC, allowing for all possible twofold dilution combinations ranging from 4- to 1/64-fold MIC combinations of each drug. The following drugs classified as β-lactams—amoxicillin, oxacillin, cefoxitin, ceftazidime, ceftaroline fosamil hydrate, imipenem, and doripenem, and β-lactamase inhibitors—sulbactam, tazobactam, clavulanic acid, avibactam, relebactam, vaborbactam, zidebactam, and nacubactam—were included. All 21 unique β-lactam + β-lactam (B + B) combinations, 28 unique β-lactamase inhibitor + β-lactamase inhibitor (BLI + BLI) combinations, and 56 unique β-lactam + β-lactamase inhibitor (1B + 1BLI) combinations were evaluated. Using *Mab* grown to exponential phase, 10^5^ CFU of *Mab* was inoculated into each well in 200 µL final volume per well of 96-well culture plate. Two wells containing Middlebrook 7H9 broth only and two wells containing broth inoculated with 10^5^ CFU of *Mab* were included as negative and positive controls, respectively, and incubated at 30°C for 72 h without shaking in accordance with the CLSI guidelines (31). Growth or lack thereof of *Mab* was assessed using the Sensititre Manual Viewbox. The FICI of drug combinations was calculated as described (27): FICI_XY_ = FICI_X_ + FICI_Y_= ([X]/MIC_X_) + ([Y]/ MIC_Y_). MIC_X_ and MIC_Y_ are MICs of drugs X and Y, respectively, when used alone. [X] and [Y] are the concentrations of the drugs X and Y, respectively, in the first well in decreasing drug concentrations where *Mab* growth is not observed. As growth is not observed in multiple wells in two axes, the mean FICI_XY_ is calculated from the FICI_XY_ from each well and reported as the final FICI_XY_.

### Determination of FICI for three drugs (2B + 1BLI or 1B + 2BLI) or four drugs (2B + 2BLI)

An identical protocol as described above for two agents was used, with the exception of combining two drugs in one axis. For instance, to determine FICIs of combinations comprising two β-lactams and one β-lactamase inhibitor (2B + 1BLI), all 120 unique combinations arising from 15 dual β-lactams (2B) that exhibited synergism (Fig. 1A) and all 8 β-lactamase inhibitors were considered. Dual β-lactams with each β-lactam at fourfold its MIC were combined. This combination and β-lactamase inhibitor at fourfold its MIC were serially diluted in the same way as described for two-drug combinations. The FICI of three drug combinations was calculated as described (27): FICI_XYZ_ = FICI_X_ + FICI_Y_ + FICI_Z_ = ([X]/MIC_X_) + ([Y]/MIC_Y_) + ([Z]/MIC_Z_).

Similarly, to determine the FICI for four drugs comprising two β-lactams and two β-lactamase inhibitors (2B + 2BLI), all 165 unique combinations arising from 15 dual β-lactams (2B) that exhibited synergism (Fig. 1A) and 11 dual β-lactamase inhibitors that exhibited synergism (Fig. 1C) were considered. Dual β-lactams with each β-lactam at fourfold its MIC were combined. Similarly, dual β-lactamase inhibitors with each β-lactamase inhibitor at fourfold its MIC were combined. The dual β-lactam and dual β-lactamase inhibitor combinations, starting at fourfold its MIC, were serially diluted in the same way as described for two-drug combinations. The FICI of four-drug combinations was calculated as described (27): FICI_WZYZ_ = FICI_W_ + FICI_X_ + FICI_Y_ + FICI_Z_ = ([W]/MIC_W_) + ([X]/MIC_X_) + ([Y]/MIC_Y_) + ([Z]/MIC_Z_). For a four-drug combination to achieve synergy, each component would have to be present at ≤1/16× their respective MICs for the net FICI to be ≤0.5.

### Fluorescent labeling of β-lactam targets

Meropenem-Cy5 (Mero-Cy5) was generated through a Cu-catalyzed azide-alkyne click reaction as previously reported (30). Bocillin FL (Invitrogen, catalog no. 13233) was purchased commercially and handled according to the manufacturer’s protocols. *Mab* 19977 whole-cell lysate (15 µg total protein) was labeled with 5 µM Mero-Cy5 or Bo FL (60 min, RT, in the dark). Labeled lysates were resolved via SDS-PAGE, fixed, and imaged on a Typhoon multimodal imager (Cytiva).

## SUPPLEMENTAL MATERIAL

Supplemental material aac.00127-25-s0001.pdf Tables S1 and S2.

ASM does not own the copyrights to Supplemental Material that may be linked to, or accessed through, an article. The authors have granted ASM a non-exclusive, world-wide license to publish the Supplemental Material files. Please contact the corresponding author directly for reuse.

## References

[1] Hamad B. 2010. The antibiotics market. Nat Rev Drug Discov 9:675–676. doi:10.1038/nrd3267 20811374

[2] Waxman DJ, Strominger JL. 1983. Penicillin-binding proteins and the mechanism of action of beta-lactam antibiotics. Annu Rev Biochem 52:825–869. doi:10.1146/annurev.bi.52.070183.004141 6351730

[3] Bush K, Bradford PA. 2020. Epidemiology of β-lactamase-producing pathogens. Clin Microbiol Rev 33:e00047-19. doi:10.1128/CMR.00047-19 32102899 PMC7048014

[4] Bush K, Bradford PA. 2016. β-lactams and β-lactamase inhibitors: an overview. Cold Spring Harb Perspect Med 6:a025247. doi:10.1101/cshperspect.a025247 27329032 PMC4968164

[5] Coyette J, Perkins HR, Polacheck I, Shockman GD, Ghuysen JM. 1974. Membrane-bound DD-carboxypeptidase and LD-transpeptidase of Streptococcus faecalis ATCC 9790. Eur J Biochem 44:459–468. doi:10.1111/j.1432-1033.1974.tb03504.x 4209348

[6] Mainardi JL, Fourgeaud M, Hugonnet JE, Dubost L, Brouard JP, Ouazzani J, Rice LB, Gutmann L, Arthur M. 2005. A novel peptidoglycan cross-linking enzyme for a β-lactam-resistant transpeptidation pathway. J Biol Chem 280:38146–38152. doi:10.1074/jbc.M507384200 16144833

[7] Dubée V, Triboulet S, Mainardi JL, Ethève-Quelquejeu M, Gutmann L, Marie A, Dubost L, Hugonnet JE, Arthur M. 2012. Inactivation of Mycobacterium tuberculosis L,D-transpeptidase LdtMt₁ by carbapenems and cephalosporins. Antimicrob Agents Chemother 56:4189–4195. doi:10.1128/AAC.00665-12 22615283 PMC3421625

[8] Kumar P, Kaushik A, Lloyd EP, Li SG, Mattoo R, Ammerman NC, Bell DT, Perryman AL, Zandi TA, Ekins S, Ginell SL, Townsend CA, Freundlich JS, Lamichhane G. 2017. Non-classical transpeptidases yield insight into new antibacterials. Nat Chem Biol 13:54–61. doi:10.1038/nchembio.2237 27820797 PMC5477059

[9] Kumar P, Chauhan V, Silva JRA, Lameira J, d’Andrea FB, Li S-G, Ginell SL, Freundlich JS, Alves CN, Bailey S, Cohen KA, Lamichhane G. 2017. Mycobacterium abscessus L,D-transpeptidases are susceptible to inactivation by carbapenems and cephalosporins but not penicillins. Antimicrob Agents Chemother 61:e00866-17. doi:10.1128/AAC.00866-17 28760902 PMC5610527

[10] Lavollay M, Fourgeaud M, Herrmann JL, Dubost L, Marie A, Gutmann L, Arthur M, Mainardi JL. 2011. The peptidoglycan of Mycobacterium abscessus is predominantly cross-linked by L,D-transpeptidases. J Bacteriol 193:778–782. doi:10.1128/JB.00606-10 21097619 PMC3021224

[11] Lavollay M, Arthur M, Fourgeaud M, Dubost L, Marie A, Veziris N, Blanot D, Gutmann L, Mainardi JL. 2008. The peptidoglycan of stationary-phase Mycobacterium tuberculosis predominantly contains cross-links generated by L,D-transpeptidation. J Bacteriol 190:4360–4366. doi:10.1128/JB.00239-08 18408028 PMC2446752

[12] Gupta R, Lavollay M, Mainardi JL, Arthur M, Bishai WR, Lamichhane G. 2010. The Mycobacterium tuberculosis protein LdtMt2 is a nonclassical transpeptidase required for virulence and resistance to amoxicillin. Nat Med 16:466–469. doi:10.1038/nm.2120 20305661 PMC2851841

[13] Kumar P, Arora K, Lloyd JR, Lee IY, Nair V, Fischer E, Boshoff HIM, Barry CE 3rd. 2012. Meropenem inhibits D,D-carboxypeptidase activity in Mycobacterium tuberculosis. Mol Microbiol 86:367–381. doi:10.1111/j.1365-2958.2012.08199.x 22906310 PMC3468717

[14] Magnet S, Bellais S, Dubost L, Fourgeaud M, Mainardi JL, Petit-Frère S, Marie A, Mengin-Lecreulx D, Arthur M, Gutmann L. 2007. Identification of the L,D-transpeptidases responsible for attachment of the Braun lipoprotein to Escherichia coli peptidoglycan. J Bacteriol 189:3927–3931. doi:10.1128/JB.00084-07 17369299 PMC1913343

[15] Magnet S, Arbeloa A, Mainardi JL, Hugonnet JE, Fourgeaud M, Dubost L, Marie A, Delfosse V, Mayer C, Rice LB, Arthur M. 2007. Specificity of L,D-transpeptidases from Gram-positive bacteria producing different peptidoglycan chemotypes. J Biol Chem 282:13151–13159. doi:10.1074/jbc.M610911200 17311917

[16] Peltier J, Courtin P, El Meouche I, Lemée L, Chapot-Chartier MP, Pons JL. 2011. Clostridium difficile has an original peptidoglycan structure with a high level of N-acetylglucosamine deacetylation and mainly 3-3 cross-links. J Biol Chem 286:29053–29062. doi:10.1074/jbc.M111.259150 21685382 PMC3190713

[17] Sanders AN, Wright LF, Pavelka MS. 2014. Genetic characterization of mycobacterial L,D-transpeptidases. Microbiology (Reading) 160:1795–1806. doi:10.1099/mic.0.078980-0 24855140 PMC4117223

[18] Wietzerbin J, Das BC, Petit JF, Lederer E, Leyh-Bouille M, Ghuysen JM. 1974. Occurrence of D-alanyl-(D)-meso-diaminopimelic acid and meso-diaminopimelyl-meso-diaminopimelic acid interpeptide linkages in the peptidoglycan of mycobacteria. Biochemistry 13:3471–3476. doi:10.1021/bi00714a008 4210702

[19] Tuomanen E, Cozens R. 1987. Changes in peptidoglycan composition and penicillin-binding proteins in slowly growing Escherichia coli*.* J Bacteriol 169:5308–5310. doi:10.1128/jb.169.11.5308-5310.1987 3312172 PMC213942

[20] Nguyen DC, Dousa KM, Kurz SG, Brown ST, Drusano G, Holland SM, Kreiswirth BN, Boom WH, Daley CL, Bonomo RA. 2021. “One-two punch”: synergistic ß-lactam combinations for Mycobacterium abscessus and target redundancy in the inhibition of peptidoglycan synthesis enzymes. Clin Infect Dis 73:1532–1536. doi:10.1093/cid/ciab535 34113990 PMC8677594

[21] Mattoo R, Lloyd EP, Kaushik A, Kumar P, Brunelle JL, Townsend CA, Lamichhane G. 2017. Ldt_Mav2_, a nonclassical transpeptidase and susceptibility of Mycobacterium avium to carbapenems. Future Microbiol 12:595–607. doi:10.2217/fmb-2016-0208 28555497 PMC5619143

[22] Story-Roller E, Maggioncalda EC, Cohen KA, Lamichhane G. 2018. Mycobacterium abscessus and β-lactams: emerging insights and potential opportunities. Front Microbiol 9:2273. doi:10.3389/fmicb.2018.02273 30319581 PMC6167491

[23] Soroka D, Dubée V, Soulier-Escrihuela O, Cuinet G, Hugonnet JE, Gutmann L, Mainardi JL, Arthur M. 2014. Characterization of broad-spectrum Mycobacterium abscessus class A β-lactamase. J Antimicrob Chemother 69:691–696. doi:10.1093/jac/dkt410 24132992

[24] Rominski A, Schulthess B, Müller DM, Keller PM, Sander P. 2017. Effect of β-lactamase production and β-lactam instability on MIC testing results for Mycobacterium abscessus. J Antimicrob Chemother 72:3070–3078. doi:10.1093/jac/dkx284 28961987

[25] Kumar G, Galanis C, Batchelder HR, Townsend CA, Lamichhane G. 2022. Penicillin binding proteins and β-lactamases of Mycobacterium tuberculosis: reexamination of the historical paradigm. mSphere 7:e0003922. doi:10.1128/msphere.00039-22 35196121 PMC8865919

[26] Dubée V, Bernut A, Cortes M, Lesne T, Dorchene D, Lefebvre AL, Hugonnet JE, Gutmann L, Mainardi JL, Herrmann JL, Gaillard JL, Kremer L, Arthur M. 2015. β-lactamase inhibition by avibactam in Mycobacterium abscessus. J Antimicrob Chemother 70:1051–1058. doi:10.1093/jac/dku510 25525201

[27] Hsieh MH, Yu CM, Yu VL, Chow JW. 1993. Synergy assessed by checkerboard. A critical analysis. Diagn Microbiol Infect Dis 16:343–349. doi:10.1016/0732-8893(93)90087-n 8495592

[28] Odds FC. 2003. Synergy, antagonism, and what the chequerboard puts between them. J Antimicrob Chemother 52:1–1. doi:10.1093/jac/dkg301 12805255

[29] Hecker SJ, Reddy KR, Totrov M, Hirst GC, Lomovskaya O, Griffith DC, King P, Tsivkovski R, Sun D, Sabet M, Tarazi Z, Clifton MC, Atkins K, Raymond A, Potts KT, Abendroth J, Boyer SH, Loutit JS, Morgan EE, Durso S, Dudley MN. 2015. Discovery of a cyclic boronic acid β-lactamase inhibitor (RPX7009) with utility vs class a serine carbapenemases. J Med Chem 58:3682–3692. doi:10.1021/acs.jmedchem.5b00127 25782055

[30] Levine SR, Beatty KE. 2021. Investigating β-Lactam drug targets in Mycobacterium tuberculosis using chemical probes . ACS Infect Dis 7:461–470. doi:10.1021/acsinfecdis.0c00809 33470787 PMC8096124

[31] CLSI. 2023. Performance Standards for Susceptibility Testing of Mycobacteria, Nocardia spp., and Other Aerobic Actinomycetes, 2nd Edition. Wayne, PA, USA CLSI supplement M24S Clinical and Laboratory Standards Institute 31339680

[32] Lefebvre AL, Dubée V, Cortes M, Dorchêne D, Arthur M, Mainardi JL. 2016. Bactericidal and intracellular activity of β-lactams against Mycobacterium abscessus. J Antimicrob Chemother 71:1556–1563. doi:10.1093/jac/dkw022 26929268

[33] Papp-Wallace KM, McLeod SM, Miller AA. 2023. Durlobactam, a Broad-Spectrum Serine β-lactamase Inhibitor, Restores Sulbactam Activity Against Acinetobacter Species. Clin Infect Dis 76:S194–S201. doi:10.1093/cid/ciad095 37125470 PMC10150275

[34] Story-Roller E, Galanis C, Lamichhane G. 2021. β-Lactam Combinations That Exhibit Synergy against Mycobacteroides abscessus Clinical Isolates. Antimicrob Agents Chemother 65:e02545-20. doi:10.1128/AAC.02545-20 33361310 PMC8097488

[35] Edoo Z, Iannazzo L, Compain F, Li de la Sierra Gallay I, van Tilbeurgh H, Fonvielle M, Bouchet F, Le Run E, Mainardi J, Arthur M, Ethève‐Quelquejeu M, Hugonnet J. 2018. Synthesis of avibactam derivatives and activity on β‐Lactamases and peptidoglycan biosynthesis enzymes of mycobacteria. Chemistry A European J 24:8081–8086. doi:10.1002/chem.201800923 29601108

[36] Meir M, Bifani P, Barkan D. 2018. The addition of avibactam renders piperacillin an effective treatment for Mycobacterium abscessus infection in an in vivo model. Antimicrob Resist Infect Control 7:151. doi:10.1186/s13756-018-0448-4 30564307 PMC6293638

[37] Soroka D, Ourghanlian C, Compain F, Fichini M, Dubée V, Mainardi JL, Hugonnet JE, Arthur M. 2017. Inhibition of β-lactamases of mycobacteria by avibactam and clavulanate. J Antimicrob Chemother 72:1081–1088. doi:10.1093/jac/dkw546 28039278

[38] Story-Roller E, Maggioncalda EC, Lamichhane G. 2019. Select β-lactam combinations exhibit synergy against Mycobacterium abscessus in vitro Antimicrob Agents Chemother 63:e02613-18. doi:10.1128/AAC.02613-18 30745389 PMC6437493

[39] Pandey R, Chen L, Manca C, Jenkins S, Glaser L, Vinnard C, Stone G, Lee J, Mathema B, Nuermberger EL, Bonomo RA, Kreiswirth BN. 2019. Dual β-lactam combinations highly active against Mycobacterium abscessus complex in vitro mBio 10:1–14. doi:10.1128/mBio.02895-18 PMC6372805 30755518

[40] Dousa KM, Kurz SG, Taracila MA, Bonfield T, Bethel CR, Barnes MD, Selvaraju S, Abdelhamed AM, Kreiswirth BN, Boom WH, Kasperbauer SH, Daley CL, Bonomo RA. 2020. Insights into the L,D-transpeptidases and D,D-carboxypeptidase of Mycobacterium abscessus: ceftaroline, imipenem, and novel diazabicyclooctane inhibitors. Antimicrob Agents Chemother 64:e00098-20. doi:10.1128/AAC.00098-20 32393499 PMC7526840

[41] Pozuelo Torres M, van Ingen J. 2024. Dual β-lactam therapy to improve treatment outcome in Mycobacterium abscessus disease. Clin Microbiol Infect 30:738–742. doi:10.1016/j.cmi.2024.03.019 38527611

[42] Moore M, Frerichs JB. 1953. An unusual acid-fast infection of the knee with subcutaneous, abscess-like lesions of the gluteal region. Journal of Investigative Dermatology 20:133–169. doi:10.1038/jid.1953.18 13035193

[43] Larsen M. 2000. Some common methods in mycobacterial genetics, p 313–320. In Hatfull Graham F., Jacobs William R. (ed), Molecular genetics of mycobacteria. American Society for Microbiology, Washington, DC.

