# Supplementary material for: Combinations comprising dual β-lactams and a β-lactamase inhibitor achieve optimal synergistic inhibition of Mycobacterium abscessus growth
Source: Antimicrob Agents Chemother. 2025 Aug 14;69(10):e00127-25. doi: 10.1128/aac.00127-25 (PMC12486838; doi:10.1128/aac.00127-25)
Supplement: Supplemental material — Tables S1 and S2. [file aac.00127-25-s0001.pdf]

SUPPLEMENTAL INFORMATION

Combinations comprising dual β-lactams and a β-lactamase inhibitor achieve optimal synergistic inhibition of *Mycobacterium abscessus* growth

Binayak Rimal[a], Yi Xie[a], Chandra M. Panthi[a], Kaylyn L. Devlin[b], Kimberly E. Beatty[b], Gyanu Lamichhane[a,c,]*

[a]Division of Infectious Diseases, Department of Medicine, School of Medicine, Johns Hopkins University, Baltimore, MD 21231, USA. [b]Department of Chemical Physiology and Biochemistry, Oregon Health Sciences University, Portland, OR 97239, USA. [c]Center for Nontuberculous Mycobacteria and Bronchiectasis, School of Medicine, Johns Hopkins University, Baltimore, MD 21231, USA.

SUPPLEMENTARY TABLE

Table S1: Minimum inhibitory concentrations (MIC) in µg/mL of drugs against *M. abscessus* ATCC 19977.

DRUG	MICs (µg/mL)
Amoxicillin	1024
Oxacillin	1024
Cefoxitin	16
Ceftazidime	512
Ceftaroline	512
Imipenem	8
Doripenem	16
Sulbactam	1024
Tazobactam	1024
Clavulanic acid	512
Avibactam	512
Relebactam	1024
Vaborbactam	512
Zidebactam	512
Nacubactam	512

MICs were determined in accordance with the Clinical and Laboratory Standards Institute guidelines (1).

Table S2: Fractional Inhibitory Concentration Index (FICI) of combinations comprising of four agents.

β-lactam1 + β-lactam2 + β-lactamase inhibitor1 + β-lactamase inhibitor2	FICI
Amoxicillin + Imipenem + Tazobactam + Avibactam	0.25
Amoxicillin + Imipenem + Sulbactam + Relebactam	0.25
Amoxicillin + Imipenem + Tazobactam + Nacubactam	0.25
Amoxicillin + Ceftaroline + Tazobactam + Avibactam	0.25
Amoxicillin + Ceftaroline + Tazobactam + Relebactam	0.25
Amoxicillin + Ceftaroline + Sulbactam + Avibactam	0.25
Amoxicillin + Ceftaroline + Sulbactam + Nacubactam	0.25
Amoxicillin + Ceftaroline + Sulbactam + Relebactam	0.25
Amoxicillin + Ceftaroline + Tazobactam + Nacubactam	0.25
Amoxicillin + Doripenem + Tazobactam + Avibactam	0.25
Amoxicillin + Doripenem + Sulbactam + Nacubactam	0.25
Amoxicillin + Doripenem + Sulbactam + Relebactam	0.25
Amoxicillin + Doripenem + Tazobactam + Nacubactam	0.25
Amoxicillin + Ceftazidime + Tazobactam + Avibactam	0.25
Amoxicillin + Ceftazidime + Tazobactam + Nacubactam	0.25
Amoxicillin + Cefoxitin + Tazobactam + Avibactam	0.25
Amoxicillin + Cefoxitin + Tazobactam + Relebactam	0.25
Amoxicillin + Cefoxitin + Sulbactam + Avibactam	0.25
Amoxicillin + Cefoxitin + Sulbactam + Nacubactam	0.25
Amoxicillin + Cefoxitin + Sulbactam + Relebactam	0.25
Amoxicillin + Cefoxitin + Tazobactam + Nacubactam	0.25
Amoxicillin + Imipenem + Tazobactam + Relebactam	0.38
Amoxicillin + Imipenem + Sulbactam + Avibactam	0.38
Amoxicillin + Imipenem + Sulbactam + Nacubactam	0.38
Amoxicillin + Ceftaroline + Tazobactam + Vaborbactam	0.38
Amoxicillin + Doripenem + Tazobactam + Relebactam	0.38
Amoxicillin + Doripenem + Sulbactam + Avibactam	0.38
Amoxicillin + Doripenem + Tazobactam + Vaborbactam	0.38
Amoxicillin + Ceftazidime + Tazobactam + Relebactam	0.38
Amoxicillin + Ceftazidime + Sulbactam + Avibactam	0.38
Amoxicillin + Ceftazidime + Sulbactam + Nacubactam	0.38
Amoxicillin + Ceftazidime + Sulbactam + Relebactam	0.38
Amoxicillin + Ceftazidime + Tazobactam + Vaborbactam	0.38
Amoxicillin + Cefoxitin + Tazobactam + Vaborbactam	0.38
Amoxicillin + Imipenem + Tazobactam + Vaborbactam	0.50
Ceftaroline + Imipenem + Tazobactam + Avibactam	0.63
Ceftaroline + Imipenem + Tazobactam + Relebactam	0.63
Ceftaroline + Imipenem + Sulbactam + Avibactam	0.63
Ceftaroline + Imipenem + Sulbactam + Nacubactam	0.63

Ceftaroline + Imipenem + Sulbactam + Relebactam	0.63
Ceftaroline + Imipenem + Tazobactam + Nacubactam	0.63
Amoxicillin + Imipenem + Tazobactam + Zidebactam	0.63
Amoxicillin + Imipenem + Sulbactam + Zidebactam	0.63
Ceftaroline + Doripenem + Tazobactam + Avibactam	0.63
Ceftaroline + Doripenem + Tazobactam + Relebactam	0.63
Ceftaroline + Doripenem + Sulbactam + Avibactam	0.63
Ceftaroline + Doripenem + Sulbactam + Nacubactam	0.63
Ceftaroline + Doripenem + Sulbactam + Relebactam	0.63
Ceftaroline + Doripenem + Tazobactam + Nacubactam	0.63
Amoxicillin + Ceftaroline + Tazobactam + Zidebactam	0.63
Amoxicillin + Cefoxitin + Tazobactam + Zidebactam	0.63
Amoxicillin + Cefoxitin + Sulbactam + Zidebactam	0.63
Cefoxitin + Ceftaroline + Tazobactam + Avibactam	0.63
Cefoxitin + Ceftaroline + Tazobactam + Relebactam	0.63
Cefoxitin + Ceftaroline + Sulbactam + Nacubactam	0.63
Cefoxitin + Ceftaroline + Sulbactam + Relebactam	0.63
Amoxicillin + Imipenem + Clavulanic acid + Sulbactam	0.75
Amoxicillin + Ceftaroline + Sulbactam + Zidebactam	0.75
Amoxicillin + Ceftazidime + Tazobactam + Zidebactam	0.75
Amoxicillin + Ceftazidime + Sulbactam + Zidebactam	0.75
Cefoxitin + Ceftaroline + Tazobactam + Clavulanic acid	0.75
Cefoxitin + Ceftaroline + Tazobactam + Nacubactam	0.75
Ceftaroline + Doripenem + Tazobactam + Vaborbactam	1.00
Amoxicillin + Doripenem + Tazobactam + Clavulanic acid	1.00
Amoxicillin + Doripenem + Tazobactam + Zidebactam	1.00
Amoxicillin + Ceftazidime + Clavulanic acid + Sulbactam	1.00
Oxacillin + Imipenem + Tazobactam + Nacubactam	1.00
Amoxicillin + Cefoxitin + Tazobactam + Clavulanic acid	1.00
Amoxicillin + Cefoxitin + Clavulanic acid + Sulbactam	1.00
Cefoxitin + Ceftaroline + Tazobactam + Vaborbactam	1.00
Cefoxitin + Ceftaroline + Tazobactam + Zidebactam	1.00
Ceftaroline + Imipenem + Tazobactam + Zidebactam	1.13
Ceftaroline + Ceftazidime + Tazobactam + Avibactam	1.13
Ceftaroline + Ceftazidime + Tazobactam + Relebactam	1.13
Ceftaroline + Ceftazidime + Tazobactam + Clavulanic acid	1.13
Ceftaroline + Ceftazidime + Sulbactam + Avibactam	1.13
Ceftaroline + Ceftazidime + Sulbactam + Nacubactam	1.13
Ceftaroline + Ceftazidime + Clavulanic acid + Sulbactam	1.13
Ceftaroline + Ceftazidime + Sulbactam + Zidebactam	1.13
Ceftaroline + Doripenem + Tazobactam + Clavulanic acid	1.13
Ceftaroline + Doripenem + Tazobactam + Zidebactam	1.13
Ceftaroline + Doripenem + Sulbactam + Zidebactam	1.13
Amoxicillin + Ceftaroline + Tazobactam + Clavulanic acid	1.13

Amoxicillin + Doripenem + Clavulanic acid + Sulbactam	1.13
Amoxicillin + Doripenem + Sulbactam + Zidebactam	1.13
Oxacillin + Imipenem + Tazobactam + Vaborbactam	1.13
Oxacillin + Imipenem + Tazobactam + Zidebactam	1.13
Oxacillin + Imipenem + Clavulanic acid + Sulbactam	1.13
Cefoxitin + Doripenem + Tazobactam + Nacubactam	1.13
Amoxicillin + Ceftaroline + Clavulanic acid + Sulbactam	1.25
Amoxicillin + Ceftazidime + Tazobactam + Clavulanic acid	1.25
Oxacillin + Imipenem + Sulbactam + Nacubactam	1.25
Ceftazidime + Imipenem + Clavulanic acid + Sulbactam	1.25
Cefoxitin + Ceftaroline + Sulbactam + Zidebactam	1.25
Cefoxitin + Doripenem + Tazobactam + Avibactam	1.25
Ceftaroline + Imipenem + Tazobactam + Clavulanic acid	1.50
Ceftaroline + Imipenem + Tazobactam + Vaborbactam	1.50
Ceftaroline + Imipenem + Clavulanic acid + Sulbactam	1.50
Amoxicillin + Imipenem + Tazobactam + Clavulanic acid	1.50
Ceftaroline + Ceftazidime + Sulbactam + Relebactam	1.50
Ceftaroline + Ceftazidime + Tazobactam + Nacubactam	1.50
Ceftaroline + Ceftazidime + Tazobactam + Vaborbactam	1.50
Ceftaroline + Ceftazidime + Tazobactam + Zidebactam	1.50
Imipenem + Doripenem + Tazobactam + Nacubactam	1.50
Imipenem + Doripenem + Tazobactam + Vaborbactam	1.50
Imipenem + Doripenem + Tazobactam + Zidebactam	1.50
Imipenem + Doripenem + Clavulanic acid + Sulbactam	1.50
Imipenem + Doripenem + Sulbactam + Zidebactam	1.50
Ceftazidime + Doripenem + Tazobactam + Clavulanic acid	1.50
Ceftaroline + Doripenem + Clavulanic acid + Sulbactam	1.50
Oxacillin + Imipenem + Tazobactam + Relebactam	1.50
Oxacillin + Imipenem + Sulbactam + Zidebactam	1.50
Ceftazidime + Imipenem + Tazobactam + Avibactam	1.50
Cefoxitin + Ceftaroline + Sulbactam + Avibactam	1.50
Cefoxitin + Ceftaroline + Clavulanic acid + Sulbactam	1.50
Cefoxitin + Doripenem + Tazobactam + Vaborbactam	1.50
Cefoxitin + Doripenem + Tazobactam + Zidebactam	1.50
Ceftaroline + Imipenem + Sulbactam + Zidebactam	2.00
Ceftazidime + Doripenem + Tazobactam + Avibactam	2.00
Ceftazidime + Doripenem + Tazobactam + Relebactam	2.00
Ceftazidime + Doripenem + Sulbactam + Relebactam	2.00
Ceftazidime + Doripenem + Tazobactam + Nacubactam	2.00
Ceftazidime + Doripenem + Tazobactam + Vaborbactam	2.00
Ceftazidime + Doripenem + Tazobactam + Zidebactam	2.00
Ceftazidime + Doripenem + Clavulanic acid + Sulbactam	2.00
Ceftazidime + Doripenem + Sulbactam + Zidebactam	2.00
Cefoxitin + Imipenem + Clavulanic acid + Sulbactam	2.00

Oxacillin + Imipenem + Sulbactam + Avibactam	2.00
Oxacillin + Imipenem + Sulbactam + Relebactam	2.00
Ceftazidime + Imipenem + Tazobactam + Relebactam	2.00
Ceftazidime + Imipenem + Tazobactam + Clavulanic acid	2.00
Ceftazidime + Imipenem + Sulbactam + Avibactam	2.00
Ceftazidime + Imipenem + Tazobactam + Nacubactam	2.00
Ceftazidime + Imipenem + Tazobactam + Vaborbactam	2.00
Ceftazidime + Imipenem + Tazobactam + Zidebactam	2.00
Ceftazidime + Imipenem + Sulbactam + Zidebactam	2.00
Cefoxitin + Doripenem + Tazobactam + Relebactam	2.00
Cefoxitin + Doripenem + Tazobactam + Clavulanic acid	2.00
Cefoxitin + Doripenem + Sulbactam + Avibactam	2.00
Cefoxitin + Doripenem + Sulbactam + Nacubactam	2.00
Cefoxitin + Doripenem + Sulbactam + Relebactam	2.00
Cefoxitin + Doripenem + Clavulanic acid + Sulbactam	2.00
Cefoxitin + Doripenem + Sulbactam + Zidebactam	2.00
Imipenem + Doripenem + Sulbactam + Avibactam	2.13
Imipenem + Doripenem + Sulbactam + Nacubactam	2.13
Cefoxitin + Imipenem + Tazobactam + Nacubactam	2.13
Cefoxitin + Imipenem + Tazobactam + Vaborbactam	2.13
Cefoxitin + Imipenem + Tazobactam + Zidebactam	2.13
Cefoxitin + Imipenem + Sulbactam + Zidebactam	2.13
Oxacillin + Imipenem + Tazobactam + Avibactam	2.13
Imipenem + Doripenem + Tazobactam + Avibactam	2.25
Imipenem + Doripenem + Tazobactam + Relebactam	2.25
Imipenem + Doripenem + Tazobactam + Clavulanic acid	2.25
Ceftazidime + Imipenem + Sulbactam + Nacubactam	2.25
Imipenem + Doripenem + Sulbactam + Relebactam	2.50
Ceftazidime + Doripenem + Sulbactam + Avibactam	2.50
Ceftazidime + Doripenem + Sulbactam + Nacubactam	2.50
Oxacillin + Imipenem + Tazobactam + Clavulanic acid	2.50
Ceftazidime + Imipenem + Sulbactam + Relebactam	2.50
Cefoxitin + Imipenem + Tazobactam + Relebactam	3.00
Cefoxitin + Imipenem + Tazobactam + Clavulanic acid	3.00
Cefoxitin + Imipenem + Sulbactam + Avibactam	3.00
Cefoxitin + Imipenem + Sulbactam + Nacubactam	3.00
Cefoxitin + Imipenem + Sulbactam + Relebactam	3.00
Cefoxitin + Imipenem + Tazobactam + Avibactam	3.50

FICI of four drug combinations comprising two β-lactams and two β-lactamase inhibitors were determined using the checkeboard assay as described (2). Combinations with fractional inhibitory concentration index (FICI) ≤ 0.5, indicating synergism, are shaded green. Culture conditions recommended for growing and testing *M. abscessus* in the Clinical and Laboratory Standards Institute guidelines were used (1).

REFERENCES

1. CLSI. 2023. Performance Standards for Susceptibility Testing of Mycobacteria, Nocardia spp., and Other Aerobic Actinomycetes, 2nd Edition. CLSI M24S2nd Editio. CLSI.

2. Hsieh MH, Yu CM, Yu VL, Chow JW. 1993. Synergy assessed by checkerboard. A critical analysis. Diagn Microbiol Infect Dis 16:343–9.
